# Primary Malignant Mixed Müllerian Mesodermal Tumor Mimicking a Rectosigmoid Carcinoma: A Case Report and Review of the Literature

**DOI:** 10.1155/2014/948908

**Published:** 2014-02-10

**Authors:** Sakshi Kapur, Levin Miles

**Affiliations:** ^1^Department of Internal Medicine, Overlook Medical Center, 99 Beauvoir Avenue, Summit, NJ 07902, USA; ^2^Division of Pathology, Overlook Medical Center, 99 Beauvoir Avenue, Summit, NJ 07902, USA

## Abstract

We report a case of a 53-year-old female who presented with chronic constipation and abdominal discomfort for six months. Her past surgical history was significant for a total abdominal hysterectomy with bilateral salpingooophorectomy, performed eight years ago, for uterine fibroids and endometriosis. Workup revealed a mass measuring 5 × 4.5 × 2 cm in the rectosigmoid colon. Patient underwent a low anterior resection and a fungating, centrally ulcerated rectosigmoid mass with a positive mesorectal margin was removed. Histopathology revealed a heterologous mixed mesodermal tumor (chondroid and osteoid elements). The epithelial component was compatible with a grade 2 endometrioid adenocarcinoma. Immunohistochemical stains were supportive, with positive expression for CK7 and ER, negative for CK20, and only very focally and weakly positive for both CDX2 and p63. Chromogranin, synaptophysin, and TTF-1 were negative. Following surgery, she was treated with five cycles of carboplatin (AUC 6) and paclitaxel (175 mg/m^2^), followed by irradiation. Twenty-six months later, patient continues to be asymptomatic and disease-free. Mixed müllerian mesodermal tumors mimicking colorectal cancer have been reported in the past. Our case highlights the rarity and the challenges encountered in diagnosing and treating these rare tumors.

## 1. Introduction

Mixed müllerian mesodermal tumors are rare tumors of uncertain origin. Although these tumors account for 2–5% of all uterine malignancies, extragenital mixed müllerian mesodermal tumors have been reported in various locations ranging from pelvic peritoneum to diaphragm peritoneum. We report a case of a 53-year-old female who presented with chronic constipation and abdominal discomfort for six months. Workup revealed a rectosigmoid mass compatible with a malignant mixed müllerian mesodermal tumor. Patient was treated with low anterior resection, five cycles of chemotherapy (carboplatin and paclitaxel), and irradiation. 

## 2. Case Report

A 53-year-old Portuguese female presented to our hospital with chronic constipation and abdominal discomfort for six months. She denied any change in her appetite or weight. Her past surgical history was significant for a total abdominal hysterectomy with bilateral salpingooophorectomy, performed eight years ago, for uterine fibroids and endometriosis. She denied any postmenopausal or gastrointestinal bleeding. Physical examination revealed an average sized female with no acute distress. Her systemic examination was unremarkable.

**Figure 1:**
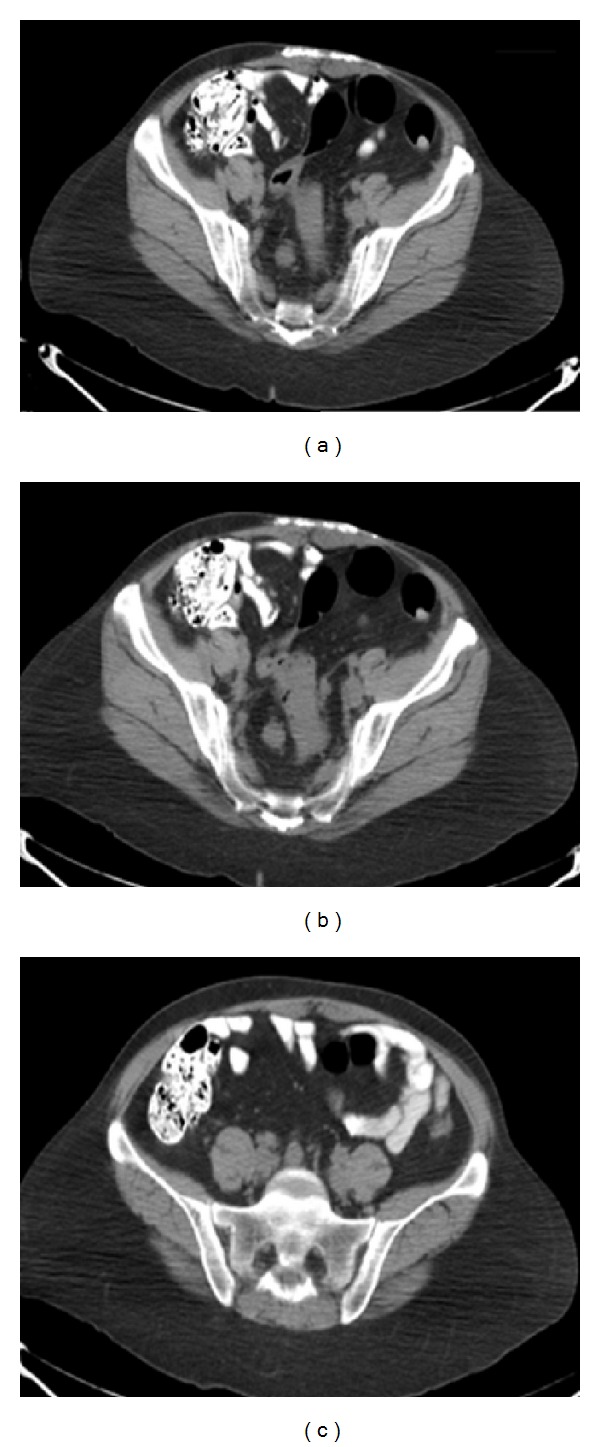
Computer tomography of the abdomen showing (a) an abnormal soft tissue density involving the sigmoid colon; (b) an enlarged lymph node right to the sigmoid colon; (c) multiple enlarged lymph nodes just below the bifurcation of the abdominal aorta into the common iliac arteries.

Laboratory workup revealed hemoglobin 13.1 g/dL (normal range: 12.5–16.0 g/dL), white blood cell count 9.75 × 10^3^/uL (normal range: 4.5–11.0 × 10^3^/uL), and platelet count 254 × 10^3^/uL (normal range: 150–450 × 10^3^/uL). Blood urea nitrogen, serum creatinine, and electrolytes were within normal limits. Computer tomography of her abdomen showed an abnormal soft tissue density involving the sigmoid colon, measuring approximately 4.7 cm in anterior-posterior dimension and 2.2 cm in maximum transverse dimension. An enlarged lymph node (right of the sigmoid colon) measuring approximately 1.6 × 1.6 cm was noted. Additional enlarged lymph nodes were seen more superiorly just below the bifurcation of the abdominal aorta into the common iliac arteries, and the largest of these was seen anterior to the sacrum measuring 1.2 × 1.1 cm. The visualized portions of the urinary bladder were within normal limits and the kidneys bilaterally demonstrated no hydronephrosis (Figure 1). Ca-125 and CEA levels were 6.4 U/mL (normal range: 0–30 U/mL) and 0.8 ug/L (normal range: 0.0–3.0 ug/L), respectively.

**Figure 2:**
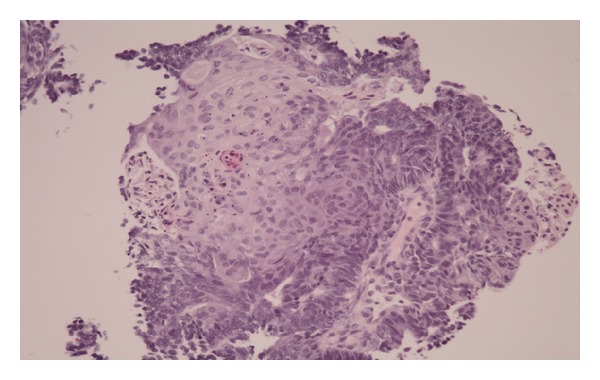
Original biopsy diagnostic of an epithelioid malignancy with variable differentiation patterns including solid areas with squamoid features and glandular areas. Diagnosis was of adenocarcinoma with squamous differentiation with qualification to exclude a carcinoma of endometrial origin.

In view of the above findings, a surgical consultation was obtained and a rectal biopsy was performed. The histopathology revealed an epithelioid malignancy with variable differentiation patterns including solid areas with basaloid and squamoid features and pseudoglandular areas. However, the typical dirty necrosis seen in conventional colonic adenocarcinomas was absent (Figure 2). Immunohistochemical stains (IHC) were positive for CK7, negative for CK20, and only very focally and weakly positive for both CDX2 (an intestinal marker) and p63 (a squamous marker). IHC for chromogranin, synaptophysin, and TTF-1 (a lung marker) were negative. These findings were compatible with an adenocarcinoma with squamous differentiation with qualification to exclude a carcinoma of endometrial origin.

**Figure 3:**
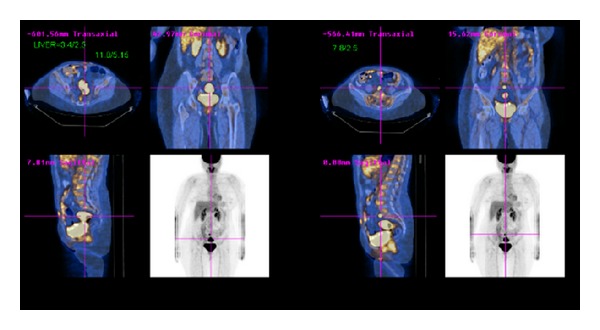
PET-CT showing a hypermetabolic rectosigmoid mass with multiple hypermetabolic enlarged lymph nodes.

A low anterior resection of the colorectal mass was planned. Whole body PET-CT confirmed a rectosigmoid mass with a maximum SUV of 12.1. An enlarged lymph node with a maximum SUV of 6 was noted right to the sigmoid colon. Multiple enlarged lymph nodes just below the bifurcation of the abdominal aorta into common iliac arteries, with a maximum SUV of 9.9, were also seen (Figure 3).

Bilateral ureteral stents were inserted prior to surgery, in order to prevent any injury to the urinary tract during dissection. Patient underwent a low anterior resection and a 5 × 4.5 × 2 cm, fungating, centrally ulcerating tumor mass with transmural involvement and a positive mesorectal margin was removed. Patient tolerated the procedure well and her postoperative period was uneventful.

**Figure 4:**
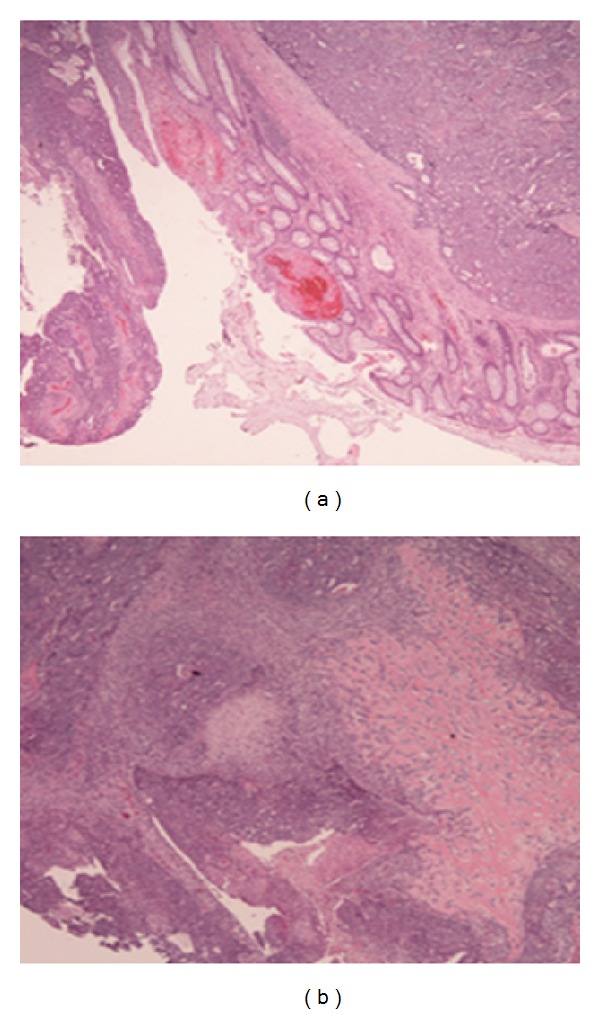
(a) H&E (2x) lower-power microscopic view of the resection specimen with an epithelioid malignancy involving the large intestine; (b) H&E (4x bone and cartilage) low-power view showing both malignant epithelial and mesenchymal elements. The epithelioid component consists of malignant glandular elements with squamous differentiation. The heterologous component (on the right) consists of a malignant osteoid.

**Figure 5:**
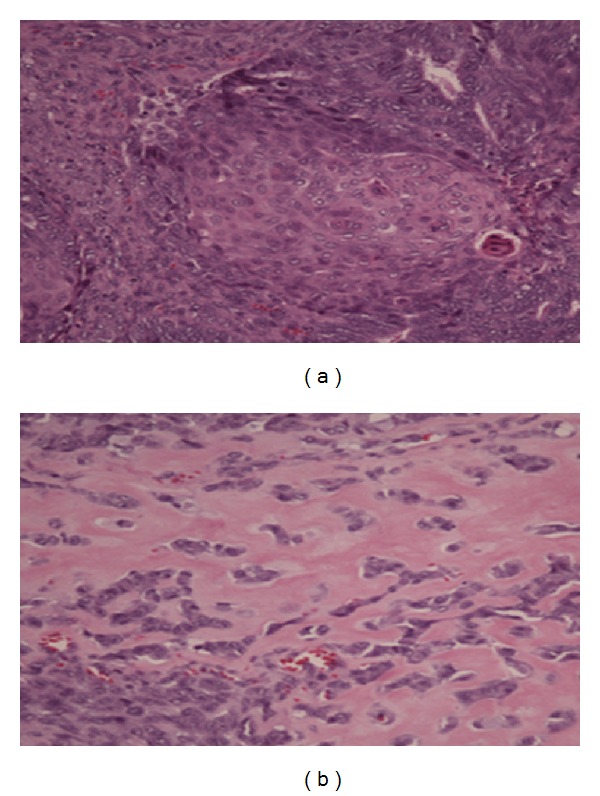
(a) (20x) H&E: showing squamous differentiation; (b) (20x) H&E: high power of malignant osteoid.

**Figure 6:**
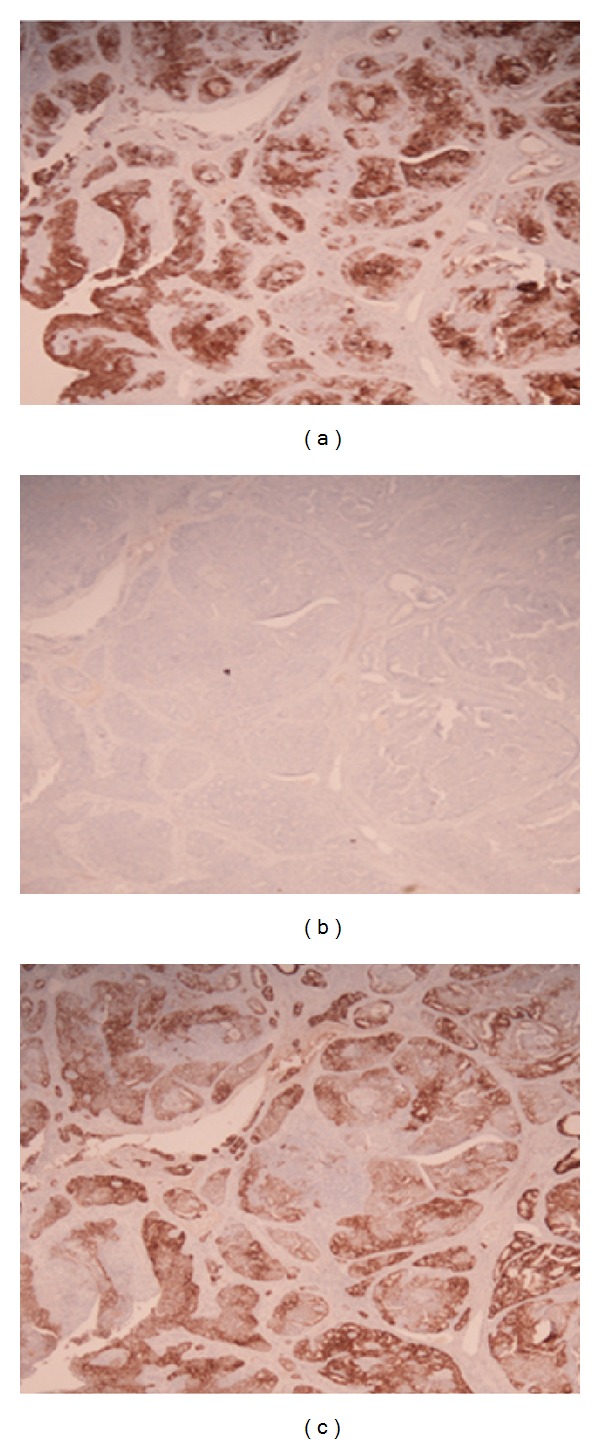
Immunohistochemistry: (a) (2x) tumor cells are positive for CK7 on the epithelioid components; (b) (2x) tumor cells show no expression for CK20; (c) (2x) tumor cells show strong diffuse expression for estrogen receptor (this immunohistochemical pattern is not consistent with colorectal primaries, but rather with müllerian-derived tumors).

The histopathology revealed findings consistent with a malignant mixed mesodermal tumor, measuring 5 × 4.5 × 2 cm, and highly suspicious of lymphovascular invasion. Although the radial margin was positive, both proximal and distal margins were negative. Positive metastasis in 3/18 lymph nodes was noted. Three separate tumor deposits were also identified (largest measuring 1.8 cm). The tumor was predominantly glandular with focal squamous differentiation, and without the typical “dirty necrosis” seen in conventional colonic adenocarcinomas. Rather, the epithelial component was that of a grade 2 endometrioid adenocarcinoma. In addition, malignant osteoid and chondroid type of heterologous elements were present. This combined morphology was of a carcinosarcoma (Figures 4 and 5). Immunohistochemistry revealed neoplastic epithelioid cells positive for ER, PAX2, PAX8, CK7, and PR focally and negative for CDX2 and CK20, supporting a müllerian primary (Figure 6).

Following surgery, patient was treated with chemotherapy and irradiation. She received paclitaxel (175 mg/m^2^) and carboplatin (AUC 6) every three weeks (a total of five cycles) followed by irradiation. Twenty-six months later, she continues to be in remission and remains asymptomatic.

## 3. Discussion

Carcinosarcoma accounts for 2–5% of all malignancies of the uterine corpus. A “mixed müllerian mesodermal tumor” (MMMT) or carcinosarcoma is composed of epithelial and mesenchymal elements, both of which are histologically malignant. These tumors most commonly arise in the uterus but can also arise from other parts of the female genital tract such as the cervix, fallopian tubes, vagina, and ovaries [1–7]. Patients usually present with a bulky pelvic abdominal mass, hemorrhagic ascites, and disseminated metastasis.

Extragenital MMMTs, although extremely rare, have been reported in the literature. Pelvic peritoneum seems to be the most common site for extragenital MMMTs (Table 1) [8–24].

Extragenital MMMTs have also been shown to arise in other sites such as the serosal surface of the colon, retroperitoneum, cul-de-sac, rectal peritoneum, anterolateral abdominal peritoneum, diaphragm peritoneum, and omentum [25–27]. Extragenital MMMTs arising in jejunal and cecal mesentery have also been reported [28, 29]. Both primary and metastatic (ovarian primary) carcinosarcomas of the spleen have been reported in the literature [30–32].

The explanation for the origin of extragenital MMMTs is generally attributed to neoplastic transformation of the multipotential coelomic mesothelium, wolffian duct remnants, or neoplastic transformation of endometriosis. A variety of müllerian malignancies have been reported to arise from, or be associated with, endometriosis including clear cell carcinoma, squamous cell carcinoma, and stromal sarcoma. Although these tumors are mostly reported in the ovaries, extraovarian malignancies have also been reported. Primary peritoneal MMMTs are also thought to arise either from foci of endometriosis, from müllerian duct remnants, or directly from the mesothelium and submesothelial mesenchyme. Lauchlan called these areas in peritoneum “secondary müllerian system” [33, 34]. It is the pluripotentiality of the peritoneum to differentiate into tumors resembling those of female genital tract that might explain the origin of these rare tumors.

Yang et al. reported a clinicopathologic study of thirteen cases of carcinoma of müllerian origin with clinical presentation mimicking primary colorectal carcinoma. The average age of these patients was 63.9 years and the major presenting symptom was a rectosigmoid mass. All tumors were surgically resected with a final diagnosis of moderately differentiated endometrioid carcinoma in six cases, mixed serous and endometrioid carcinoma in four cases, malignant mixed müllerian tumor in two cases, and undifferentiated carcinoma in one case. In nine out of thirteen cases, foci of endometriosis were identified adjacent to or within the tumor, and one case had endosalpingiosis. All except two had involvement of the colorectal mucosa [35]. Slavin et al. reported a clinical and pathological study of six cases of endometriosis-associated intestinal tumors [36].

Extragenital MMMTs have been associated with various gynecologic malignancies (synchronous or metachronous) of the primary müllerian system. Arora et al. reported a case of malignant MMMT arising in the broad ligament with a synchronous ovarian and endometrial carcinoma in a 76-year-old female [37]. Huang et al. reported a case of malignant MMMT arising from the mesorectum with a synchronous ovarian malignancy in a 50-year-old female [38]. MMMTs have also been associated with other primary tumors such as ovarian thecoma and serous adenocarcinoma [39, 40]. El-Jabbour et al. reported a case of extragenital MMMT with a synchronous colonic adenocarcinoma [41]. 

Carcinosarcoma or MMMT shares risk factors with endometrial carcinoma, but the influence of these factors is weaker than with carcinoma. MMMTs are mostly seen in postmenopausal women in the fifth and sixth decades of life, with risk factors such as obesity, nulliparity, and exogenous estrogen exposure. A history of prior pelvic irradiation has been reported in 7–37% of cases in the earlier literature, for the treatment of benign conditions such as abnormal uterine bleeding and, more recently, following treatment for cervical cancer. Carcinosarcoma may also develop after long-term treatment with tamoxifen for breast cancer. Hubalek et al. reported a case of uterine carcinosarcoma in a patient after treatment with tamoxifen for two years [42]. MMMTs have also been associated with autoimmune manifestations such as myasthenia gravis and thrombocytopenic purpura [43].

Both epithelial and mesenchymal elements in a carcinosarcoma are malignant. Often there is a sharp demarcation between the epithelial and mesenchymal elements. Most frequently the epithelial component is a poorly differentiated serous carcinoma. Rarely, squamous cell carcinoma is the sole epithelial component. Other epithelial patterns encountered are mucinous carcinoma, clear cell carcinoma, or undifferentiated carcinoma. Cokelaere et al. reported a case of MMMT in jejunal mesentery with prominent neuroendocrine differentiation [29]. The heterologous elements most commonly seen are rhabdomyosarcoma, chondrosarcoma, osteosarcoma, and liposarcoma (in order of decreasing frequency). Other rare elements encountered include neuroectodermal tissue, yolk sac tumor (associated with elevated alpha-fetoprotein), and melanocytic and neuroendocrine elements. Various immunohistochemical stains can be used to support the diagnosis of a carcinosarcoma. MMMTs are usually positive for CK7 and ER and negative for CK20 and CDX2. In addition, positivity for PR, CA 125, or WT-1 might be helpful in making a definitive diagnosis. In a study by Loy et al. out of the 165 ovarian tumors, 86% of the endometrioid ovarian tumors and none of the colorectal carcinomas were CK7+/CK20−, whereas 96% of the colorectal carcinomas and none of the endometrioid tumors were CK7−/CK20+ pattern [44]. However, some microsatellite instability high colorectal adenocarcinomas can be CK7+/CK20− negative. Therefore, additional stains with ER and CDX2 can be used to support the diagnosis. 

Extragenital MMMTs have a poorer prognosis as compared to their uterine counterparts. In a study by Garamvoelgyi et al., the median postoperative survival time in patients with extragenital MMMTs arising in pelvic peritoneum was fourteen months [13]. The mainstay of treatment for MMMTs is surgery, but most tumors are metastatic at the time of presentation, making debulking difficult. Various chemotherapeutic agents have been tried in the past. Platinum-based chemotherapy and ifosfamide were found to be superior to doxorubin for treatment of uterine carcinosarcomas [45, 46]. The combination was also found to be superior to ifosfamide alone for advanced, persistent, or recurrent disease [47, 48]. Barakat et al. studied prognostic factors in thirty patients with ovarian MMMTs. All patients were treated with chemotherapy (cisplatin and doxorubicin) following surgery. The median survival time for these patients was 10.6 months, while those with only homologous stromal elements showed an improved survival rate [49]. Ko et al. reported a case of primary peritoneal MMMT treated with surgery, chemotherapy (cisplatin and ifosfamide), and irradiation [50]. Peters et al. reported a high response rate in patients with uterine stromal sarcomas and mixed mesodermal tumors with cisplatin and adriamycin [51]. Our patient was treated with low anterior resection followed by chemotherapy and irradiation. She received a total of five cycles of carboplatin and paclitaxel, followed by radiation therapy. Twenty-six months later, she continues to be in remission. A combination of carboplatin and paclitaxel has been used successfully in the past for treating MMMTs [22, 52]. Combination of platinum-based chemotherapy and paclitaxel appears to be superior to platinum-based chemotherapy and ifosfamide in terms of toxicity and patient tolerance. Therefore, use of platinum-based chemotherapy and paclitaxel should be encouraged in patients with malignant MMMTs.

## 4. Conclusion

MMMTs are rare tumors of uncertain origin. MMMTs mimicking colorectal cancer have been reported in the past, most commonly from endometriosis. Our case highlights the importance of correct diagnosis and the challenges encountered in the management of these rare tumors.

## Figures and Tables

**Table 1:** Cases of MMMTs reported in the pelvic peritoneum and cul-de-sac.

Author	Age of the patient	Year reported	Site of extragenital MMMT
Ober and Black [8]	74 Y/F	1955	Pelvic peritoneum
Marchevsky et al. [9]	40 Y/F	1982	Cul-de-sac peritoneum
Campins et al. [10]	58 Y/F	1986	Pelvic peritoneum
Chen and Wolk [11]	52 Y/F	1988	Pelvic peritoneum
Solis et al. [12]	54 Y/F	1991	Cul-de-sac peritoneum
Garamvoelgyi et al. (reported 3 cases) [13]	60 Y/F	1994	Pelvic peritoneum, cul-de-sac, and uterine subserosa
	64 Y/F		
	84 Y/F		
Mira et al. [14]	62 Y/F	1991	Pelvic peritoneum
Ergeneli et al. [15]	80 Y/F	1997	Pelvic peritoneum
Rose et al. [16]	57 Y/F	1997	Cul-de-sac peritoneum
Shintaku and Matsumoto [17]	51 Y/F	2001	Retroperitoneum and lateral pelvic wall
Sumathi et al. (reported two cases) [18]	87 Y/F	2002	Pelvic peritoneum and omentum
	77 Y/F		
Dincer et al. [19]	50 Y/F	2002	Pelvic peritoneum
Ko et al. [20]	45 Y/F	2005	Pelvic peritoneum and cul-de-sac
Hussein et al. [21]	65 Y/F	2009	Pelvic peritoneum
U$\tilde{\text{n}}$a et al. [22]	45 Y/F	2009	Pelvic peritoneum
Naniwadekar et al. [23]	76 Y/F	2009	Pelvic peritoneum
Kurshumliu et al. [24]	72 Y/F	2011	Pelvic peritoneum
